# Cases of Cerebro-Spinal Disease in This City during the Months of February, March and April, 1872

**Published:** 1872-04-01

**Authors:** N. S. Davis


### Original Communications.

### CASES OF CEREBRO-SPINAL DISEASE IN THIS CITY DURING THE MONTHS OF FEBRUARY, MARCH AND APRIL, 1872. REMARKS ON THE TREATMENT OF THE SAME.

(*Read to the Chicago Medical Society, April* 15th, 1872.)

BY N. S. DAVIS, M.D., ETC.

Both personal observation and the statistics of mortality furnished by the Board of Health, show an unusual prevalence of cerebro-spinal disease of a serious nature, in this city during the past two months. For instance, the certificates of death returned to the health office, show 13 deaths from cerebro-spinal meningitis in February, 44 in March, and 22 during the first week in April. This not only indicates an unusual prevalence of cerebro-spinal disease, in the city, but also that it is rapidly increasing. The first case that attracted my attention was that of a young man employed as a clerk in a store, who sent for me on the 1st day of February. He was boarding at 810 Michigan avenue.

He had been attacked two days previous with chilliness and a severe pain in the head, for which he went to a physician who gave him some cathartic medicine. It operated freely on the bowels, but afforded no relief to his head. I found him in a reclining position; some flush of redness in his face, and an expression of suffering; averse to free motions, especially of the head; some general increase of sensibility or hyperæsthesia; temperature moderately increased; pulse 90 per minute, firm, and moderately full; respirations 20, and a little unsteady; tongue covered with a white coat, but moist; urine free; abdomen natural; mind despondent; and complaining of an intense pain, mostly in the lower part of the anterior region of the head, and extending at times to the occiput. The temperature of those regions of the head and the pulsation in the arteries was greater than in the other parts of the body. The pupils were small, with slight photophobia. The assemblage of symptoms was such as caused me to think there was positive inflammatory hyperæmia in the base of the brain.

The following day the pain was less intense, but in all other respects the symptoms were unchanged, except the addition of slight stiffness of the muscles of the neck, and complete aphasia. The patient understood whatever was said to him; but, in reply, repeated a number of meaningless words, mostly monosyllables, and often appeared vexed that those around him could not understand him. He was now moved to the house of a friend on Cottage Grove avenue. There, for three days, he claimed to be each day better. He recovered the power of expressing his thoughts, and had less pain in his head. But his neck remained stiff; his pulse variable; mind more dull and wandering, especially during the night. There was less febrile heat; the tongue more clean; the urine more abundant; and he insisted on sitting in his chair and taking some food every day. On the night of the 6th, his symptoms became more aggravated; his head was more retracted; mind more dull; pupils considerably dilated; pulse slow and intermitting; respirations very variable; and urine more scanty. On the 8th there was evident hemiplegia, with divergence of one eye; and entire coma followed, ending in death on the morning of the 11th. No *post mortem* examination was allowed. Since the 1st of February, a period of 72 days, there have occurred in my own practice 40 cases, sufficiently well characterized to render the diagnosis reasonably certain.

I have seen several other cases in consultation with other practitioners during the same period of time. Of the 40 cases here alluded to, 21 were in the South Division, 15 in the West, and four in the North Division of the city. Six were adults between the ages of 20 and 30 years; ten were between 5 and 15 years; and 24 were between 6 months and 5 years of age. Of the whole number 7 have died, 27 recovered, and 6 remain under treatment. Of the latter, one will probably soon terminate fatally, and the other five have a fair prospect of recovery. Of seven deaths, 3 were of adults over 20 years of age; 1 was 5

years, 1 was 3 years, and the remaining 2 six and seven months respectively.

The duration of the disease has varied much. Of the seven fatal cases, one continued 12 days, one 7 days, one 5 days, one 6 days, one 4 days, one 28 days, and one only 20 hours. Three of these cases were brought under treatment at the commencement of the attack; the other four were seen so late as to admit of only from one to three visits each. The cases that recovered have required treatment from one to four weeks each.

Four-fifths of the cases included in this report have occurred among the poor and laboring classes of people, and in a territory or part of the city bounded by East and West Harrison street on the north, south by Twenty-sixth street, east by State street, and west by Centre avenue. Yet one of the most rapidly fatal cases in the list was a little German boy five years of age, living on Wabash avenue, near Fourteenth street, who died within 20 hours from the attack.

In many instances only one child or member of a family has suffered from the disease, while in a few cases three or four in the same family have been prostrated within 24 or 36 hours. In one small house on the rear end of a lot on State street, north of Sixteenth, three children were attacked within the same 24 hours; another was attacked in the house on the front end of the same lot; another in the adjoining house south; and two others in the care of another physician in the adjoining house north. I have been unable to gather any evidences of the contagiousness or communicability of the disease. The cases have varied much, both in the severity and variety of symptoms, and yet have preserved enough of uniformity to identify them as belonging to one group, and dependent on some common pathological conditions. For instance, in all the cases the access of the disease is sudden or abrupt. They all give evidence, at first, of unusually severe pain in the head, with very variable neuralgic pains in distant parts, especially in the abdomen, thighs, and legs; and in from one to three days rigidity of the neck, with some retraction of the head and general hyperæsthesia sufficient to cause even the youngest child to manifest signs of distress on being touched or moved. In nearly all the cases there has been during the first twelve hours active vomiting, increased by raising the head to the erect position, and in some co-incident purging. These gastric and intestinal symptoms have seldom continued beyond the first one or two days. The temperature is generally increased, especially in the back of the head; the pulse frequent and firm; respirations increased in frequency, and, in most cases, panting like one excessively fatigued from severe exercise; face flushed, and expression excited and anxious at first, but subsequently dull, with dilatation of pupils; urine generally scanty and high-colored, but in some cases abundant throughout the whole duration of the disease; tongue covered with a white fur; mouth moist; and after the first one or two days, bowels inclining to constipation, with the abdomen flaccid and entirely free from tympanites. About one-third of the cases present some red, erythematic spots on the skin between the third and seventh days of the disease. These spots vary much in size and number, as well as in shade of color. In the milder cases they are bright red, and often so few in number as to attract no attention unless looked for particularly, and in others they are so numerous as to create the impression that the case may be one of scarlatina. In the more severe cases the spots are darker in color, larger in size, and in two cases they were accompanied by tumefaction from subcutaneous infiltration, as in erysipelas. In a young woman who died on the fifth day after the attack, but whom I did not see until the day previous to her death, there were numerous large purple hemorrhagic spots on the lower extremities, and an oblong, elevated, purplish red spot from one to two inches long and from half to three quarters of an inch wide on the front part of each ankle and the outer face of each wrist. The head was held rigidly to one side, eyes divergent, pupils dilated, and mind entirely unconscious. In the majority of cases, however, I failed to discover any special eruptions or spots on the surface. Nearly all the cases manifested during their progress paroxysms of excited delirium; and in the children, the first turns of vomiting would be followed by protracted turns of wild screeching and crying, and sometimes trembling as if under the influence of terrible fright. In only four cases were there general convulsions, three of which died, and one recovered.

In regard to the actual pathology or nature of this form of disease, there is still much obscurity. That it differs from *simple* inflammation of the brain and its membranes, is evident both from the symptoms during life, and the post mortem appearances after death. I have had the privilege of making only one *post mortem* examination this season. It was a case that died in the Mercy Hospital — an adult, brought in already unconscious, with rigidity of the muscles of the neck, and all the evidences of cerebro-spinal disease strongly marked. He died on the third day after admission, being about one week after the attack. Autopsy revealed a few ounces of reddish serum between the arachnoid and pia mater, and in the lateral ventricles, with the most intense and beautiful injection or turgescence of the vessels of the pia mater covering the base of the brain, medulla oblongata and upper of the spinal cord. The vessels of the brain substance were also fuller than natural, but there was no exudation of lymph or plastic material, and no other morbid appearance apparent to the eye. In a case recently alluded to by the editor of the Buffalo *Medical and Surgical Journal*, the autopsy revealed no morbid appearances in the brain or its membranes.

Most of the autopsies reported have given serous, sero-sanguineous, and sero-purulent effusions in moderate quantity, and vascular turgescence, with only slight appearances of plastic exudation. As a general rule, the more rapid the progress of the case — that is, the earlier the patient dies — the less are the visible *post mortem* changes. I have been led to regard the disease as consisting in an exaltation of the susceptibility or irritability of the structure of the cerebro-spinal axis, including the whole base of the brain, with diminished tonicity or contractility of the blood-vessels. If the alteration of the property of susceptibility is intense and extends directly to the center of the excito-motory system, it cuts short life very speedily, sometimes in a few hours, without leaving visible alterations in the brain or its membranes. But if the morbid action be less intense, or involve less directly the chief excito-motory center in the medulla oblongata, life may be prolonged until either recovery takes place or the vascular engorgement ends in suffusion of serum, etc.

*Treatment:* The first few cases coming under my care were treated with leeches to the temples and mastoid spaces; cold applications to the head; a mild cathartic and full doses of the bromide of potassa aided by hydrate of chloral to procure rest at night. After two or three days iodide of potassa was added to the bromide; counter-irritation applied to the neck. The results of this kind of treatment were not satisfactory.

The cephalalgia, muscular rigidity, etc., continued, and though the febrile action was less, there was no sign of convalescence. The first case died at the end of ten or eleven days. Another, a girl about 11 years of age, had lain with the head firmly retracted to a right angle with the shoulders; the hearing dull; mind wandering; sleepless; and often screeching with pain, for more than a week; and the foregoing remedies, with the addition of alterative doses of calomel and Dover's powder at night, had exerted no marked change in the progress of the case.

Recollecting that the Calabar bean had been used with apparent success in tetanus, and that I had used it with benefit in several cases of muscular rigidity from irritation of the nervous centers, I made the following prescription:

℞ Tinct. Calabar bean, ℥j.

Fl. Ext. Ergot, ℥jss.

Mix. Gave half a teaspoonful every two hours, and omitted all other medicines. At the end of twenty-four hours the patient was found simply more quiet, the pulse a little slower, and the respiration more regular. The treatment was continued without change. At the end of the second day the patient had ceased to complain of pain in her head, had slept several times for half an hour to an hour; the retraction of the head was decidedly less; mind was clear; the mouth moist; and the urine free; but she seemed feeble. More simple nourishment was ordered, and the medicine continued at intervals of three hours. The case continued steadily to improve; and on the fifth day after the commencement of the use of the Calabar bean and ergot, the muscular rigidity had entirely ceased, and the patient was convalescent, though emaciated and feeble. The medicine was continued three times a day several days longer, and a combination of extract of malt and the compound syrup of hypophosphites, in addition to nourishment, were given to aid in restoring nutrition and strength. Since that time I have used the Calabar bean, either alone or in combination with ergot, in nearly all the cases that have come under my care, and certainly with more apparent effect in controlling the disease than by any other remedies I have tried. The following is a brief sketch of one of the more recent cases:

Mr. A——, residing at No. 40 Brown st., on returning from his work at evening, was suddenly attacked with chilliness and violent pain in the forehead, temples, and occiput. This was soon followed by febrile reaction and vomiting. Several messages were sent to me, but I did not see him until the afternoon of the following day, when he was found in bed, with the head drawn firmly backward; face flushed; head hot; expression anxious; pupils nearly natural; pulse 100 per minute, soft; respiration hurried and irregular; mouth moist, but tongue covered with a white fur; urine scanty; bowels quiet; but promptly rejecting by vomiting whatever drink was given him, and making efforts to vomit whenever he was raised up; his mind wandering, and almost constantly crying out with the distracting pain in his head. Cloths wet in cold water had been kept on his head; and I directed the same to be continued, and immediately ordered the two following prescriptions:

℞ Tinct. Calabar bean, ℥ jss.
Fl. Ext. Ergot, ℥ jjss.

Mix. One teaspoonful to be given every two hours, in half a tablespoonful of water.

℞ Carbolic acid crystals, 6 grs.
Glycerin, ℥ ss.
Tinct. gelseminum, ℥ ss.
Water, ℥ jjj.

Mix. One teaspoonful to be given every two hours, alternately with the other; making the medicines come only one hour apart.

Directions were given to have the medicines and drinks given without raising the patient's head from the pillow, to avoid the danger of vomiting. The directions were faithfully executed, and the following day all the symptoms were moderately improved. The treatment was continued with no change except to extend the time between the doses to three hours instead of two.

The same treatment was continued four days, lengthening the interval between the doses of medicine one hour each day. On the fifth day, finding the pain in the head, the muscular rigidity, and the febrile symptoms entirely relieved, the carbolic acid mixture was omitted, the Calabar bean and ergot continued three times a day, and a saline laxative ordered to move the bowels. The patient continued to improve, and was up and dressed, apparently entirely convalescent, in eight days. If our views in regard to the essential nature of the morbid action constituting this form of disease are correct, we may expect such remedial agents as have the power to diminish *excitability*, and at the same time increase the vascular tonicity, to exert the most favorable influence over the active stages of its progress. Such are the Calabar bean, cannabis indica, gelseminum, ergot, etc. If the disease co-exists with the prevalence of erysipelas in the same community, it is probable that the free use of the sulphite of soda in addition to other remedies would be beneficial. I have noticed in several of the patients coming under my care a tendency in the disease to assume a chronic form. The constant pain in the head, the muscular rigidity, and the general febrile symptoms, gradually disappear during the first week or ten days; but the patients have continued pale, weak, subject to transient but severe neuralgic pains, ever changing in their locality, but most frequent at the head of the gastrocnemii muscles, the abdomen and the head; a very peevish, fretful condition of mind; variable appetite; and disturbed sleep. In two or three cases of this kind, a mixture of the tincture of Calabar bean and camphorated tincture of opium, given each morning, noon, and tea-time, and a moderately full dose of Dover's powder and quinine at bedtime, appeared to produce very decided and permanent relief.

In the active stage of the disease I have not found either opiates or quinine to produce any favorable effects. I have thus given, as briefly as possible, simply the results of my observations in regard to the prevalent cerebro-spinal disease, which may be said to constitute a moderate epidemic.

